# Unveiling the multidimensional cytokine landscape in acute pancreatitis

**DOI:** 10.3389/fimmu.2026.1888076

**Published:** 2026-08-11

**Authors:** Jakub W. Kosidło, Justyna Dorf, Joanna Kamińska, Olga M. Koper-Lenkiewicz, Małgorzata żaczek, Przemysław Saniuk, Karolina Zimnoch, Anatol Panasiuk, Joanna Matowicka-Karna

**Affiliations:** 1 Department of Clinical Laboratory Diagnostics, Medical University of Bialystok, Bialystok, Poland; 2 Department of Gastroenterology, Hepatology and Internal Medicine, Voivoship Hospital in Bialystok, Białystok, Poland; 3 Department of Histopathology and Cytology, Voivoship Hospital in Bialystok, Białystok, Poland; 4 Medical University of Bialystok, Bialystok, Poland

**Keywords:** acute pancreatitis, cytokine profiling, diagnostic biomarkers, IFN-γ, IL-2Rα

## Abstract

In acute pancreatitis (AP), sudden epigastric pain is accompanied by intense release of inflammatory mediators that rapidly recruit and activate innate immune cells. Pro-inflammatory cytokines (e.g., IL-6, IL-1β, TNF-α) play a key role in amplifying inflammation and may contribute to systemic complications, while anti-inflammatory cytokines (e.g., IL-10) modulate this response. Therefore, the aim of the study was to evaluate cytokines in patients with AP. We measured circulating cytokines at a single time point in 50 AP patients and 40 healthy controls using multiplex ELISA kit (Bio-Plex Pro™ Human Cytokine Screening Panel). Statistically significant increases were observed for IL-1α, IL-1β, IL-6, IL-10 and other cytokines (IL-2Rα, IFN-γ, IL-1Rα, IL-16, IL-2, MIF, IFN-α, IL-5, IL-4, IL-3, IL-12[p40], IL-12[p70], IL-17, LIF, IL-18, IL-15, IL-13; all q ≤ 0.05), alongside decreases in TNF-β (p = 0.00550, q = 0.00600) and IL 9 (p = 0.01983, q = 0.02070). IL 2Rα (AUC = 0.918) and IFN-γ (AUC = 0.907) demonstrated high discriminatory power for AP and remained significant after FDR correction (p < 0.0001, q < 0.0001). Correlation analysis linked IL-6, IL-16, and IL-1Ra with hepatic steatosis, IL-18 and IL-1Ra with alcohol use disorder and revealed an inverse relationship between IL-1β levels and severity of pancreatic necrosis. The cytokine profile reflects concurrent activation of pro inflammatory pathways, compensatory anti-inflammatory mechanisms, and early lymphocytic suppression. IL-2Rα and IFN-γ appear to be sensitive early diagnostic biomarkers, and the inverse IL-1β necrosis relationship suggests that early IL-1β signaling may facilitate efficient tissue clearance. Multi cytokine profiling may improve early risk stratification and monitoring of immunological dynamics.

## 1 Introduction

Acute pancreatitis (AP) is one of the most common causes of emergency hospitalizations for gastroenterological reasons (1). The ethology of the disease is varied and includes primarily gallstones and alcohol abuse. Less frequently, AP is caused by metabolic, drug-induced, iatrogenic, or idiopathic factors. Currently, a systematic increase in the number of AP cases is observed in developed countries (2). It is estimated that the annual incidence is approximately 30–40 cases per 100,000 people. Mortality may range from 1 to 5%; however, in severe forms of the disease it is significantly higher and reaches 30-60% (3).

The pathogenesis of AP exhibits a complex character. A key stage is the premature activation of pancreatic enzymes, e.g. trypsinogen to trypsin, which contributes to the damage of acinar and ductal cells. Uncontrolled breakdown is accompanied by the release of molecules from the DAMP – (damage-associated molecular pattern) family, which include, among others, nuclear chromatin fragments, histones or membrane proteins (4). Upon recognition of damage signals by PRR receptors on effector cells, activation of two main inflammatory pathways: NFκB (Nuclear factor kappa-light-chain-enhancer of activated B cells) and MAPK (Mitogen-Activated Protein Kinase) (5). Activated signal cascades intensify the transcription and secretion of pro-inflammatory cytokines and chemokines, which leads to the migration of leukocytes to the site of primary damage (5). The inflammatory reaction can be local, limited to the tissues of the pancreas and its surroundings, or generalized, involving the activation of immune cells throughout the body (5–7). This leads to a broad production of inflammatory and anti-inflammatory mediators. Both types of reactions can develop in parallel, and their intensity reflects the dynamics of the inflammatory process, determining the further course of the disease and prognosis (6, 7).

The central element of the inflammatory response in AP is the cytokine interaction. It includes both pro-inflammatory and anti-inflammatory mediators (8). Cytokines are responsible for regulating the activation of leukocytes. The early non-specific response is initiated mainly by IL1α, IL1β, TNFα as well as MIF, which stimulate neutrophils and intensify their migration to tissues (9, 10). Interferons, especially IFNα2 and IFNγ, activate macrophages and NK cells, increasing their ability to eliminate damaged cells (11, 12). IL6 and IL18 strengthen the inflammatory response through further activation of monocytes and dendritic cells (13). In turn, IL10 and IL1ra perform a regulatory function, limiting excessive leukocyte activation (14–16). Together they form a dynamic signal network, which shapes the intensity of the non-specific response and thereby modulates the multifactorial systemic response (1). A disturbance in the balance between these mediators favors the development of the systemic inflammatory response syndrome (SIRS) and organ failure (17). Existing studies point to an important role of interleukins, such as IL6 and IL10 as well as alpha tumor necrosis factor (TNF-α) in the course of AP (8). It has been shown that their concentrations are elevated already at the time of hospital admission. These changes may precede the development of systemic complications (18). They can occur in both the early and distant phases. In the short term, they concern mainly local changes within the pancreas and peripancreatic tissues. In a longer perspective, they can lead to persistent disorders of pancreatic function, covering both exocrine and endocrine function (6).

There is a small number of studies assessing interactions between pro- and anti-inflammatory cytokines; therefore, the aim of this study was a comprehensive evaluation of the inflammatory cytokine profile in patients with acute pancreatitis compared to a healthy control group (19–26). The study aimed to identify a characteristic pattern of cytokine changes reflecting the early activation of the inflammatory response in AP.

## 2 Materials and methods

The study was conducted in accordance with the Declaration of Helsinki and approved by the Bioethics Committee of Medical University of Bialystok, Poland, permission number APK.002.13.2025. All participants gave their written consent to participate in the study for studies involving humans and were informed about the purpose of the study.

### 2.1 Study group

The study group consisted of 50 patients (28 women, 22 men) aged 22 to 75, hospitalized at the Gastroenterology Department of the Jędrzej Śniadecki Provincial Hospital in Białystok from 1 February 2025 to 29 December 2025. Inclusion required meeting the 2014 Atlanta Consensus criteria for acute pancreatitis, defined as a threefold increase in pancreatic enzymes, severe epigastric pain, and characteristic imaging changes. Disease severity was classified according to the Revised Atlanta Classification (2012) as mild, moderately severe, or severe (27). We excluded patients with concurrent conditions, such as malignancies, chronic pancreatitis, or other inflammatory and autoimmune diseases. Pancreatic necrosis and cystic changes were initially assessed by bedside ultrasound. In patients with suspected severe disease, contrast-enhanced computed tomography (CT) was performed according to the hospital protocol. During hospitalization, pancreatic morphology was monitored with regular bedside ultrasound examinations.

### 2.2 Control group

The control group included 40 healthy patients from the occupational medicine clinic, comprising 24 women and 16 men. We screened them to ensure they had no comorbidities, active infections, or chronic inflammatory conditions. Their ages ranged from 25 to 62 years, closely matching the study group.

### 2.3 Material, sample preparation and examination

Blood samples collected for routine laboratory testing served as the primary material for our research. We collected these samples within 24 hours after patients were transferred to the ward from the Emergency Department. Blood was drawn without anticoagulants, following a protocol that required centrifugation after complete clotting but within one hour of collection. The resulting serum was immediately frozen at -80 °C.

Prior to cytokine analysis, samples were thawed gradually: first for 12 hours at -20 °C, followed by 6 hours at 4 °C until fully thawed. Once at room temperature, the samples were analyzed using the Bio-Plex Pro Human Cytokine Screening Panel (48-Plex; Bio-Rad Laboratories, Cat. No. 12007283). Although the assay allows simultaneous measurement of 48 immune mediators, including cytokines, chemokines, and growth factors, the present study focused on a predefined panel of 24 cytokines involved in the regulation of inflammatory and immune responses. The analyzed cytokines were: IL-1α, IL-1β, IL-1Rα, IL-2, IL-2Rα, IL-3, IL-4, IL-5, IL-6, IL-9, IL-10, IL-12 (p40), IL-12 (p70), IL-13, IL-15, IL-16, IL-17, IL-18, IFN-α2, IFN-γ, LIF, MIF, TNF-α, and TNF-β. Chemokines and growth factors included in the multiplex panel were outside the scope of the present analysis.

The analysis was performed using the Bio-Plex Multiplex system based on the Luminex xMAP technology. The Bio-Plex technology operates on the principles of the ELISA method. Antibodies targeting specific biomarkers form covalent bonds with magnetic beads. In the next stage, magnetic beads react with the samples containing these biomarkers. The sample is rinsed to remove unbound protein. Eventually, a biotinylated detection antibody is added to produce a sandwich compound. The final product (complex) is obtained by adding a streptavidin phycoerythrin (SA-PE) conjugate. The results are read in the Bio-Plex 200 system. The efficacy of the applied method is comparable to that of a standard ELISA assay.

### 2.4 Statistical analysis

All statistical analyses were performed using Python 3.12 (NumPy, Pandas, SciPy, Statsmodels, Scikit-learn). Data visualization was generated using Matplotlib and Seaborn. Scripted analytical framework ensured consistency and reproducibility across all procedures. Continuous variables were reported as medians. Normality of biomarker distributions was assessed using the Shapiro-Wilk test. Given the observed deviations from normality and heteroscedasticity, non-parametric tests were employed throughout. Pairwise comparisons between two groups were conducted using the two-sided Mann-Whitney U test. To control for multiple testing in confirmatory analyses, the Benjamini-Hochberg false discovery rate (FDR) procedure was applied, with statistical significance defined as *q* < 0.05. The magnitude of intergroup differences was quantified using Cliff’s delta and the Hodges-Lehmann estimator, with 95% confidence intervals derived via bootstrap resampling.

Correlation analyses were performed to explore potential relationships among cytokines and between cytokines and clinical parameters. Spearman’s rank correlation coefficients (ρ) were calculated for all analyte pairs. Given the exploratory and hypothesis-generating nature of this matrix analysis, raw (unadjusted) *p*-values were reported to maintain sensitivity for weak-to-moderate associations and to avoid overcorrection. Statistical significance for exploratory correlations was set at *p* < 0.05. This approach preserves the exploratory character of the findings while remaining fully aligned with the FDR-corrected confirmatory analyses.

The diagnostic performance of individual biomarkers was assessed using receiver operating characteristic (ROC) curve analysis. The area under the curve (AUC) was interpreted as follows: 0.50-0.70 (limited), 0.70-0.90 (moderate), and >0.90 (high). Optimal cut-off values were determined using the Youden index.

## 4 Results

### 4.1 Characteristics of the study group

The study population consisted of 50 patients, comprising 28 females (56%) and 22 males (44%). Etiological factors were distributed as follows: alcohol consumption (32%) and cholelithiasis (32%), with the remaining 36% attributed to other or unknown origins. Finally, obesity was present in 13 patients (26%), and clinical deterioration was noted in 9 cases (18%) during the first day of hospitalization. Routine laboratory parameters, including C-reactive protein (CRP), white blood cell (WBC) count, and platelet (PLT) levels, Elevated CRP levels (>10 mg/dL) were detected in 35 individuals (70%), while WBC counts exceeding 10 × 10³/μl were observed in 12 patients (24%). Platelet counts remained within normal limits for 38 patients (76%); abnormal values were recorded in the remaining cases, specifically thrombocytopenia in 10 patients (20%) and thrombocytosis in 2 patients (4%). All data presented in **Table 1**.

**Table 1:** Characteristic of the study group.

Parameter (Full name)	N (%)
Gender	
Female	28 (56%)
Male	22 (44%)
Etiology	
Alcohol	**16 (32%)**
Gallstones	**16 (32%)**
Other/Unknown	**18 (36%)**
Pancreas in imaging tests	
non-enlarged	**25 (50%)**
enlarged	**13 (26%)**
cystic lesion	**9 (18%)**
nectoric lesion	**3 (6%)**
**Obesity**	**13 (26.0%)**
**Non-obesity**	**37 (74%)**
White blood cell count	
<10 * 10^3^/μl	38 (76%)
>10 * 10^3^/μl	12 (24%)
Platelet count	
<150* 10^3^/μl	10 (20%)
150-350* 10^3^/μl	38 (76%)
>350* 10^3^/μl	2 (4%)
C-reactive protein	
<10 mg/dl	**15 (30%)**
>10 mg/dl	**35 (70%)**
Haemoglobin	
>12g/dl	**2 (4%)**
12–16 g/dl	**29 (58%)**
<12 g/dl	**19 (38%)**
Amylase	
25-125	**15 (30%)**
125-375	**20 (40%)**
>375	**15 (30%)**
Lipase	
<60	**12 (24%)**
60-180	**12 (24%)**
>180	**26 (52%)**

### 4.2 Analysis of cytokine profile in patients with acute pancreatitis and healthy controls

**Figure 1:**
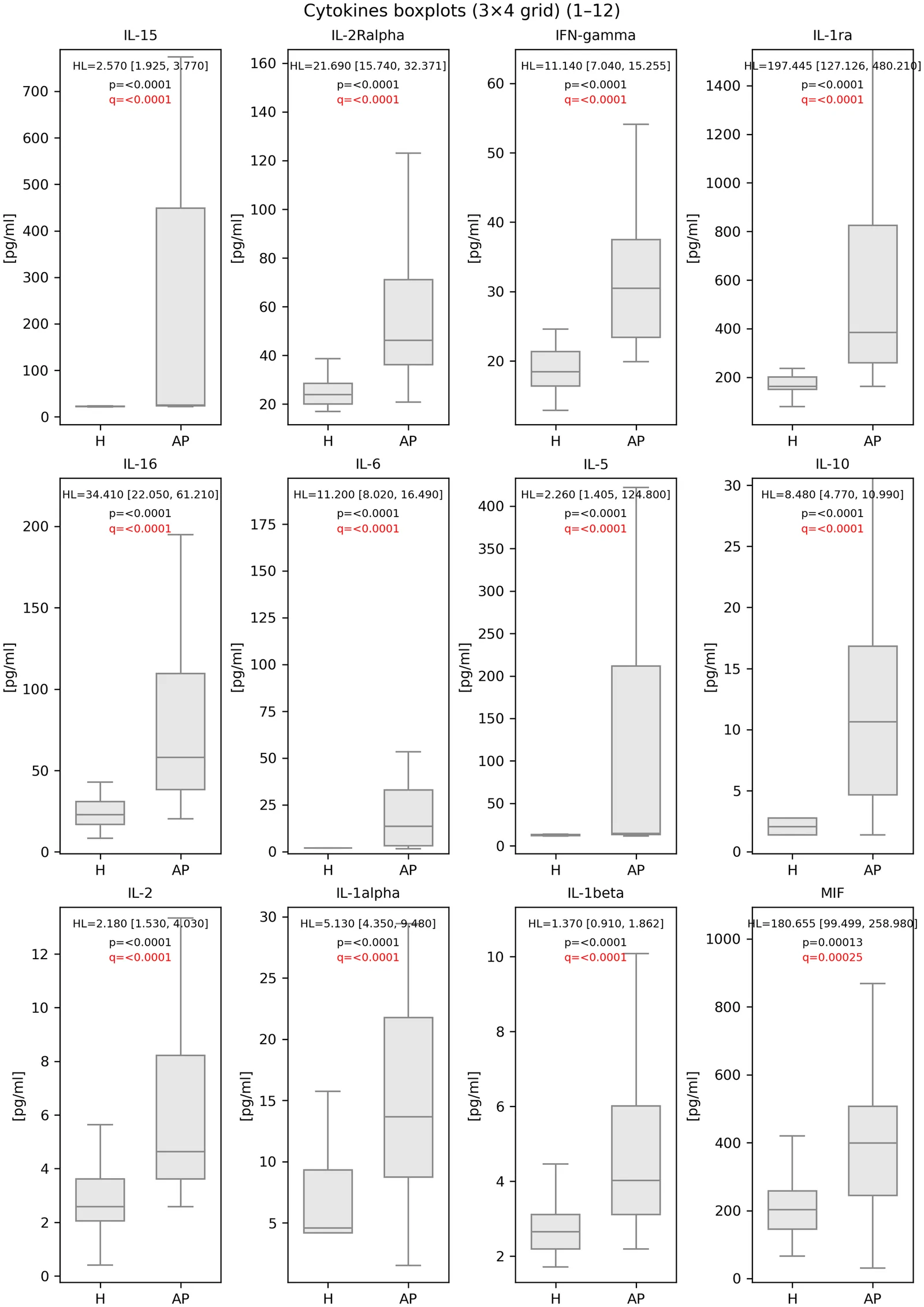
Comparison of cytokine concentrations between the control group (H) and patients with acute pancreatitis (AP).

**Figure 2:**
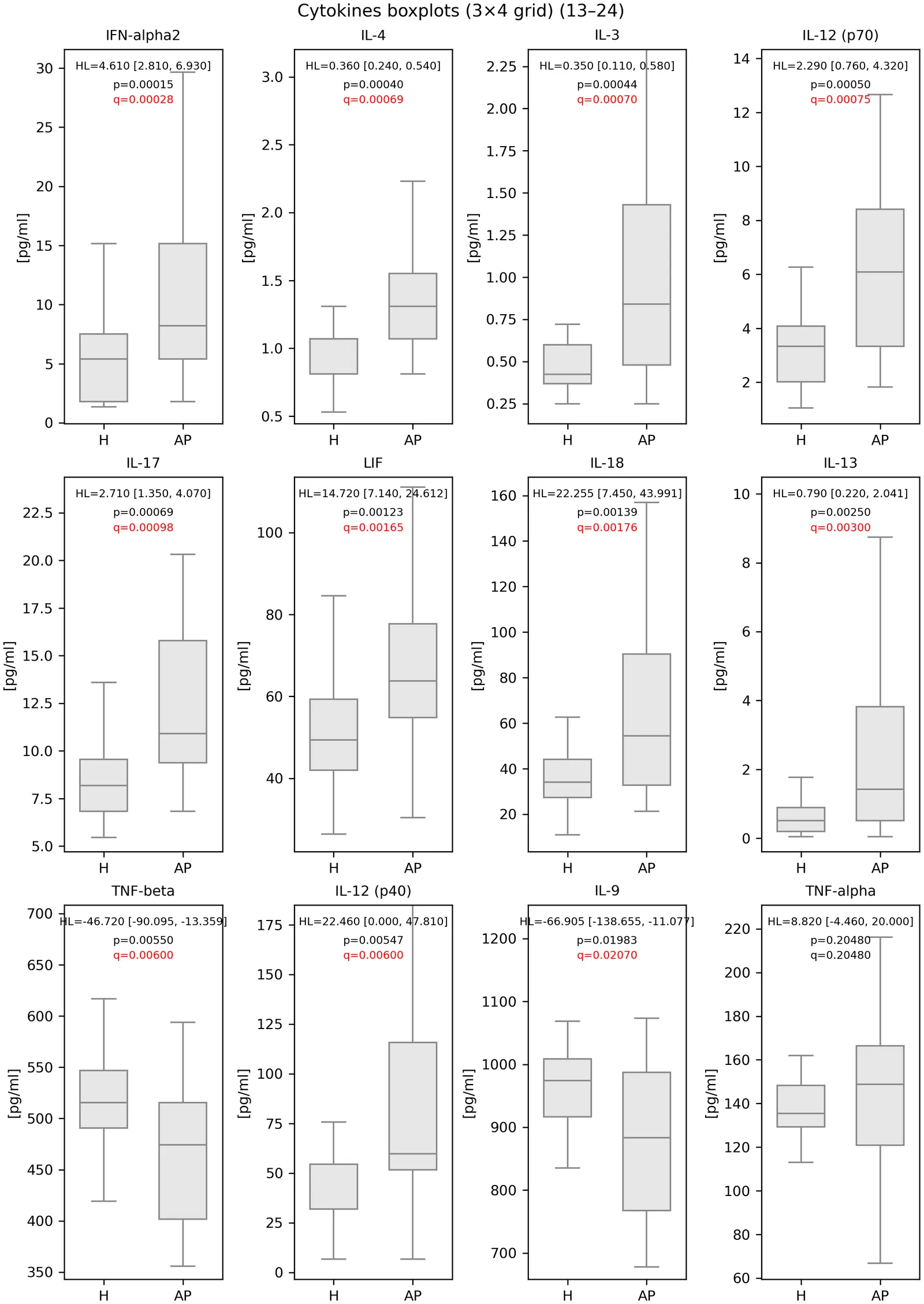
Comparison of cytokine concentrations between the control group (H) and patients with acute pancreatitis (AP).

Circulating cytokine levels differed between patients with acute pancreatitis (AP) and healthy controls (**Table 2**; **Figures 1**, **2**). After calculating correction for multiple testing, statistically significant differences were identified for 23 of the 24 analyzed cytokines. Most of these changes were associated with moderate or large effect sizes.

**Table 2:** Comparison of cytokine concentrations between the control group (H) and patients with acute pancreatitis (AP).

Marker	Median AP	Q1, Q3 AP	IQR AP	Median H	Q1, Q3 H	IQR H	MWU p	FDR q	Cliff δ	HL	HL CI
IL-15	24.980	(23.410, 448.960)	425.550	22.220	(21.640, 22.930)	1.290	<0.0001	<0.0001	0.876	2.570	[1.925, 3.770]
IL-2Rα	46.155	(36.110, 71.075)	34.965	23.800	(20.098, 28.485)	8.387	<0.0001	<0.0001	0.836	21.690	[15.740, 32.371]
IFN-γ	30.450	(23.410, 37.510)	14.100	18.440	(16.400, 21.357)	4.957	<0.0001	<0.0001	0.814	11.140	[7.040, 15.255]
IL-1Rα	384.955	(260.252, 825.570)	565.317	162.330	(150.320, 201.140)	50.820	<0.0001	<0.0001	0.793	197.445	[127.126, 480.210]
IL-16	57.975	(38.250, 109.635)	71.385	22.880	(16.873, 31.015)	14.142	<0.0001	<0.0001	0.797	34.410	[22.050, 61.210]
IL-6	13.625	(3.208, 33.002)	29.795	2.000	(2.000, 2.000)	0.000	<0.0001	<0.0001	0.713	11.200	[8.020, 16.490]
IL-5	14.615	(13.357, 211.540)	198.183	12.520	(11.838, 13.122)	1.285	<0.0001	<0.0001	0.710	2.260	[1.405, 124.800]
IL-10	10.640	(4.652, 16.840)	12.188	2.055	(1.370, 2.740)	1.370	<0.0001	<0.0001	0.675	8.480	[4.770, 10.990]
IL-2	4.640	(3.620, 8.210)	4.590	2.590	(2.060, 3.620)	1.560	<0.0001	<0.0001	0.671	2.180	[1.530, 4.030]
IL-1α	13.670	(8.748, 21.780)	13.033	4.590	(4.190, 9.320)	5.130	<0.0001	<0.0001	0.612	5.130	[4.350, 9.480]
IL-1β	4.020	(3.110, 6.008)	2.898	2.650	(2.190, 3.110)	0.920	<0.0001	<0.0001	0.615	1.370	[0.910, 1.862]
MIF	398.520	(244.987, 507.240)	262.253	202.950	(145.543, 257.885)	112.342	0.00013	0.00025	0.562	180.655	[99.499, 258.980]
IFN-α2	8.220	(5.410, 15.150)	9.740	5.410	(1.810, 7.518)	5.707	0.00015	0.00028	0.549	4.610	[2.810, 6.930]
IL-4	1.310	(1.070, 1.550)	0.480	1.070	(0.810, 1.070)	0.260	0.00040	0.00069	0.512	0.360	[0.240, 0.540]
IL-3	0.840	(0.480, 1.430)	0.950	0.425	(0.370, 0.600)	0.230	0.00044	0.00070	0.511	0.350	[0.110, 0.580]
IL-12 (p70)	6.080	(3.340, 8.410)	5.070	3.340	(2.020, 4.080)	2.060	0.00050	0.00075	0.508	2.290	[0.760, 4.320]
IL-17	10.900	(9.380, 15.787)	6.407	8.190	(6.830, 9.550)	2.720	0.00069	0.00098	0.494	2.710	[1.350, 4.070]
LIF	63.790	(54.830, 77.730)	22.900	49.370	(41.930, 59.343)	17.413	0.00123	0.00165	0.472	14.720	[7.140, 24.612]
IL-18	54.485	(32.835, 90.302)	57.467	34.035	(27.365, 44.060)	16.695	0.00139	0.00176	0.469	22.255	[7.450, 43.991]
IL-13	1.425	(0.510, 3.815)	3.305	0.510	(0.203, 0.890)	0.688	0.00250	0.00300	0.440	0.790	[0.220, 2.041]
TNF-β	474.140	(401.812, 515.433)	113.620	515.425	(490.790, 546.670)	55.880	0.00550	0.00600	-0.408	-46.720	[-90.095, -13.359]
IL-12 (p40)	59.850	(51.733, 115.860)	64.127	32.030	(32.030, 54.490)	22.460	0.00547	0.00600	0.401	22.460	[0.000, 47.810]
IL-9	883.630	(767.298, 986.995)	219.697	974.495	(916.803, 1008.788)	91.985	0.01983	0.02070	-0.342	-66.905	[-138.655, -11.077]
TNF-alpha	148.760	(120.915, 166.360)	45.445	135.450	(129.317, 148.207)	18.890	0.20480	0.20480	0.187	8.820	[-4.460, 20.000]

Values in red indicate statistical significance (p or q < 0.05). AP, Acute Pancreatitis; H, healthy; MWU, Mann Whitney-U test; FDR, False Discovery Rate; HL, Hodges–Lehmann estimator; CI, Confidence Interval.

The clearest differences involved IL-2Rα, IFN-γ, IL-1Rα, IL-16, and IL-6. IL-2Rα concentrations were higher in AP patients (Me = 46.155) than in controls (Me = 23.800; p < 0.0001, q < 0.0001). The effect size was very large (δ = 0.836), with an HL estimate of 21.690 (95% CI: 15.740–32.371). IFN-γ showed a comparable pattern, with higher median values in AP (Me = 30.450) in comparison with the control group (Me = 18.440; p < 0.0001, q < 0.0001; δ = 0.814; HL = 11.140 [7.040–15.255]). Furthermore, increases were also observed for IL-1Rα, IL-16, and IL-6 in the study group. Median IL-1Rα was decreased in healthy individuals (Me = 162.330) compared to patients with AP (Me = 384.955; δ = 0.793; HL = 197.445 [127.126–480.210]). Higher IL-16 concentration was observed in the AP group compared to the control group (Me = 57.975 vs. Me = 22.880; δ = 0.797; HL = 34.410 [22.050–61.210]). IL-6 showed a lower level in the healthy control group (Me = 2.000) than in the AP group (Me = 13.625; δ = 0.713; HL = 11.200 [8.020–16.490]). All three cytokines remained significant after FDR correction and were characterized by large effect sizes. Higher concentrations in AP were additionally noted for IL-10, IL-2, IL-1α, IL-1β, MIF, IFN-α2, IL-5, IL-4, IL-3, IL-12 (p70), IL-17, LIF, IL-18, IL-15, IL-13, and IL-12 (p40) (all q ≤ 0.05). The magnitude of these differences varied between markers. However, the direction of change was consistent across the group. By comparison, TNF-β and IL-9 concentrations were lower in AP patients. TNF-β concentration reached lower median values of 515.425 in the control group compared to 474.140 in the AP group (p = 0.00550, q = 0.00600; δ = −0.408; HL = −46.720 [−90.095, −13.359]). Similar observations were made for IL-9 concentration (AP Me = 883.630 vs. H Me = 974.495; p = 0.01983, q = 0.02070; δ = −0.342; HL = −66.905 [−138.655, −11.077]). No significant difference was found for TNF-α. Median values were comparable between AP patients and healthy individuals (p = 0.20480, q = 0.20480).

### 4.3 Comparison of cytokine profile in patients with acute pancreatitis according to etiology and severity

**Figure 3:**
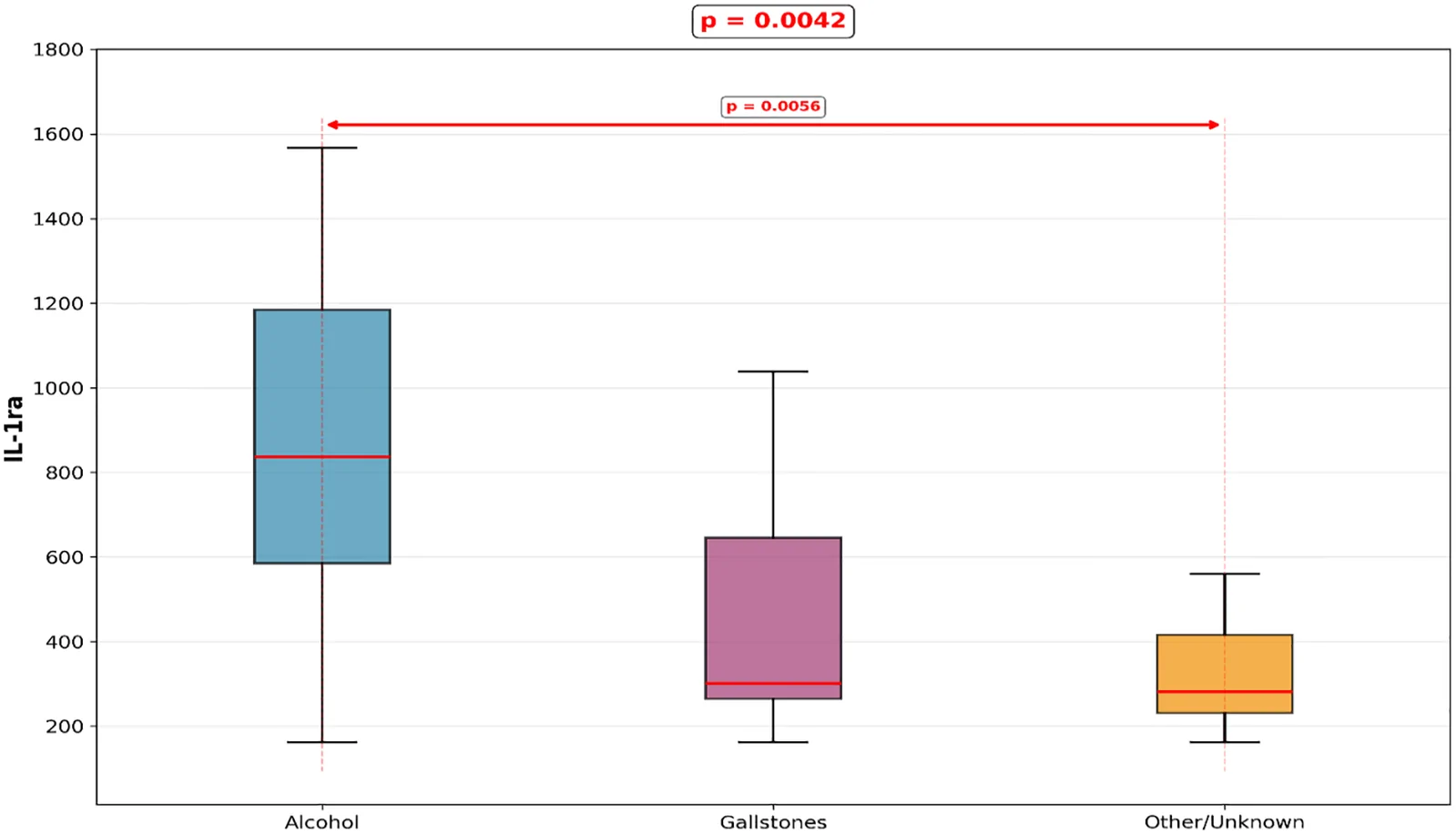
Comparison of IL-1rα concentration between patients with different etiology of acute pancreatitis: alcohol, presence of gallstones or other/unknown.

The Kruskal-Wallis test confirmed significant differences across the three groups (patients with alcohol, gallstones and unknown etiology) for IL-1ra (p = 0.0042) and IL-18 (p = 0.0197) (**Table 3**; **Figures 3**, **4A**). *Post-hoc* testing showed that the patients with alcohol etiology differed significantly from the group with unknown etiology (**Table 4**). For IL-1ra, the adjusted p-value was 0.0056 with a large effect size (Cliff’s Delta = 0.709). IL-18 showed a comparable result with an adjusted p-value of 0.0197 and a large effect size (Cliff’s Delta = 0.621). No significant differences were found for the Gallstones group after correction. The analysis of cytokine concentrations in different degrees of severity of acute pancreatitis did not show statistically significant differences.

**Table 3:** *Post-hoc* analysis (Dunn’s test with Bonferroni correction).

Cytokine	Comparison	U statistic	P-value	P (adj)	Cliff’s delta	Effect size	HL estimator
IL-1ra	Alcohol vs Gallstones	131.00	0.0181	0.0543	0.550	large	481.94
IL-1ra	Alcohol vs Other/Unknown	155.50	0.0019	0.0056	0.709	large	516.06
IL-1ra	Gallstones vs Other/Unknown	108.50	0.4085	1.0000	0.192	small	45.50
IL-18	Alcohol vs Gallstones	123.50	0.0483	0.1449	0.462	medium	37.93
IL-1ra	Alcohol vs Other/Unknown	147.50	0.0066	0.0197	0.621	large	large
IL-18	Gallstones vs Other/Unknown	99.00	0.7158	1.0000	0.088	no	4.80

**Figure 4A:**
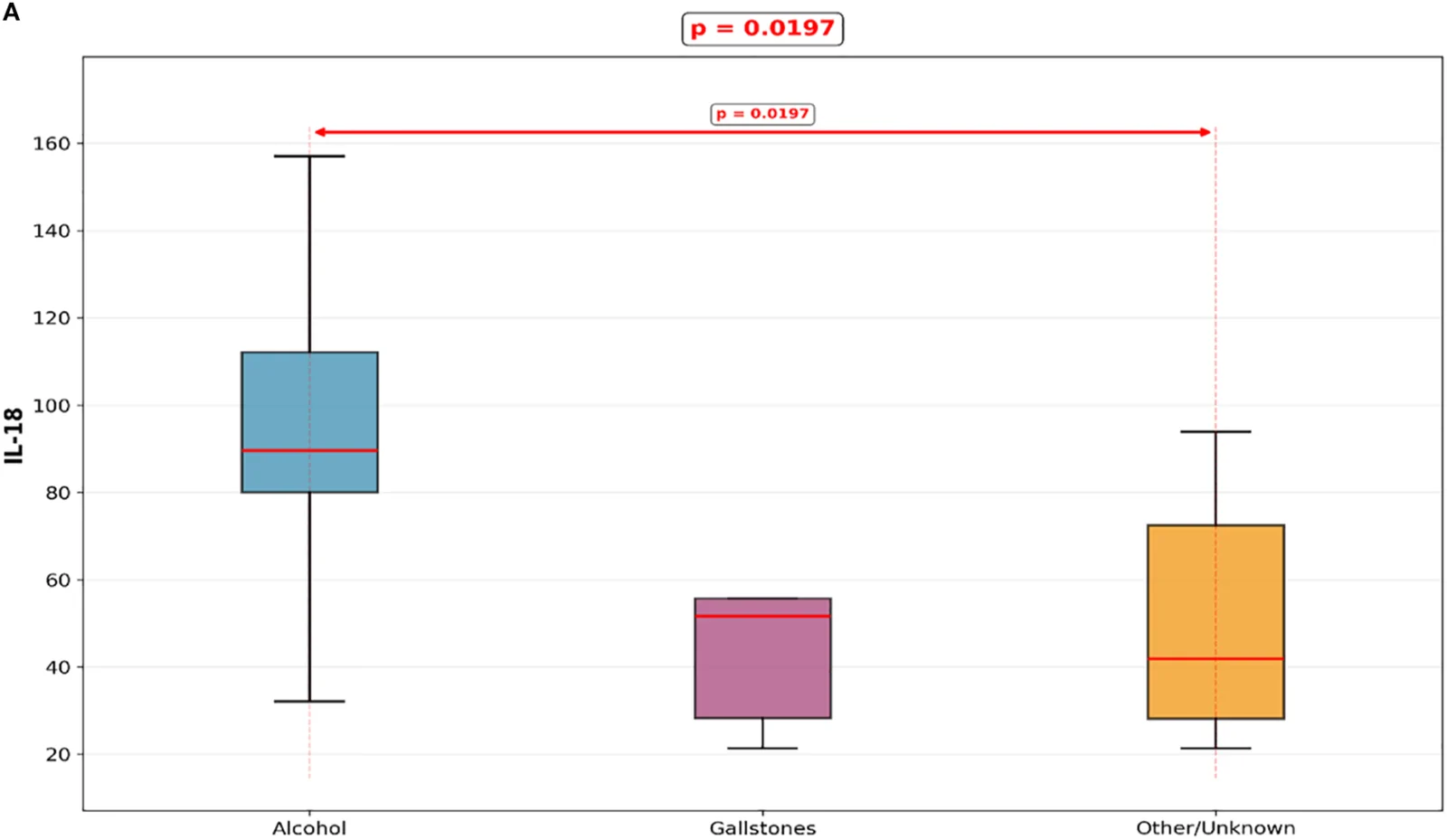
Comparison of IL-18 concentration between patients with different etiology of acute pancreatitis: alcohol, presence of gallstones or other/unknown.

**Figure 4B:**
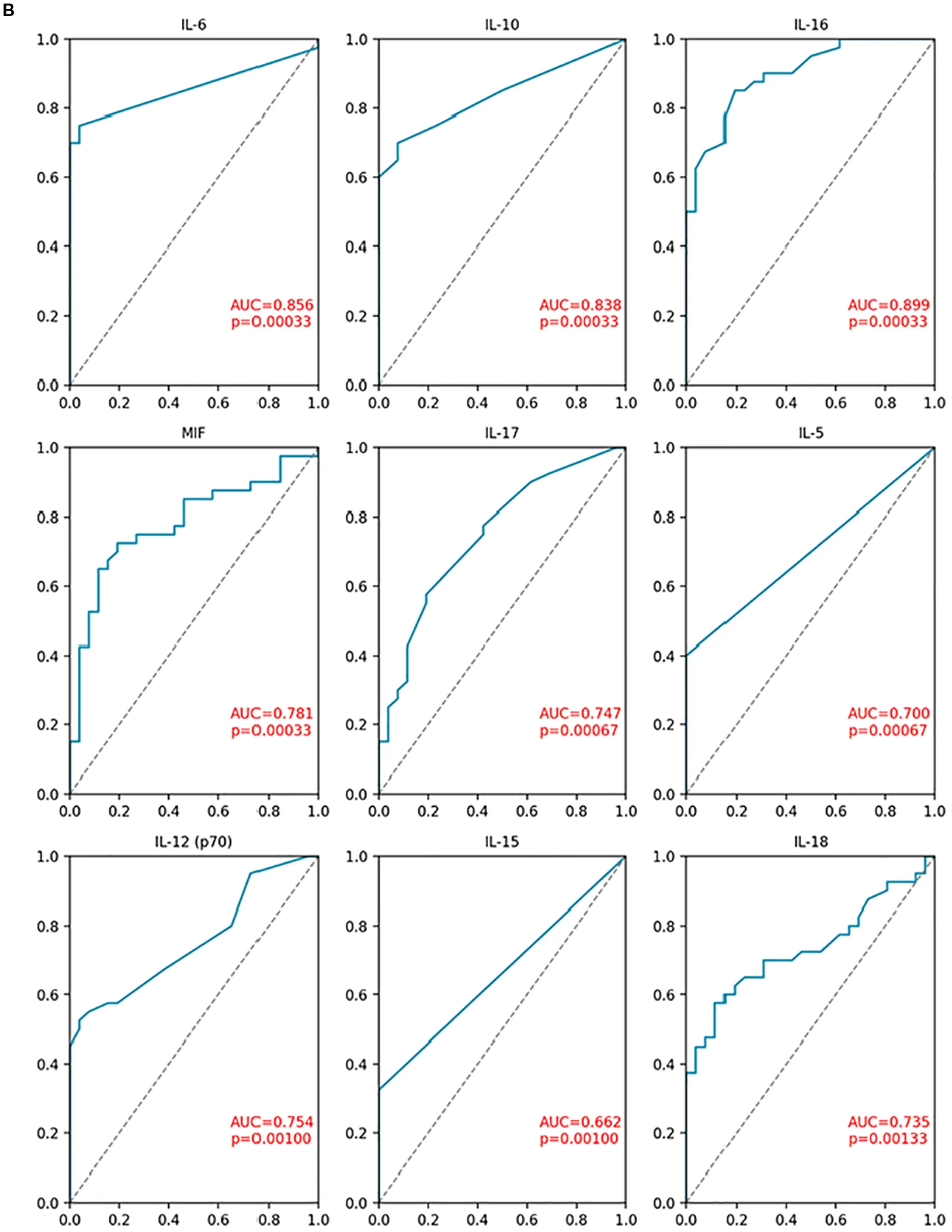
Receiver operating characteristic (ROC) analysis of cytokines of patients with acute pancreatitis and the control group.

**Table 4:** Receiver operating characteristic (ROC) analysis of cytokines of patients with acute pancreatitis and the control group.

Marker	AUC	AUC 95% CI	p	FDR q	Sensitivity	Specifity
IFN-α2	0.775	0.655–0.879	<0.0001	<0.0001	0.700	0.731
IFN- γ	0.907	0.824–0.973	<0.0001	<0.0001	1.000	0.692
IL-1α	0.806	0.694–0.903	<0.0001	<0.0001	0.525	0.923
IL-1β	0.808	0.705–0.902	<0.0001	<0.0001	0.725	0.769
IL-1rα	0.897	0.806–0.973	0.0003	0.0006	0.825	0.923
IL-2	0.836	0.733–0.923	0.0003	0.0006	0.825	0.692
IL-2Rα	0.918	0.843–0.980	0.0003	0.0006	0.900	0.846
IL-3	0.755	0.638–0.865	0.0003	0.0006	0.550	0.923
IL-4	0.756	0.642–0.873	0.0003	0.0006	0.625	0.808
IL-6	0.856	0.771–0.927	0.0003	0.0006	0.750	0.962
IL-10	0.838	0.744–0.928	0.0003	0.0006	0.700	0.923
IL-16	0.899	0.821–0.963	0.0003	0.0006	0.850	0.808
MIF	0.781	0.665–0.887	0.0003	0.0006	0.650	0.885
IL-17	0.747	0.629–0.856	0.0006	0.001	0.575	0.808
IL-5	0.700	0.625–0.774	0.0006	0.001	0.400	1.000
IL-12 (p70)	0.754	0.637–0.858	0.001	0.0014	0.525	0.962
IL-15	0.662	0.595–0.733	0.001	0.0014	0.325	1.000
IL-18	0.735	0.609–0.843	0.0013	0.0018	0.575	0.885
LIF	0.736	0.612–0.853	0.00167	0.00210	0.600	0.769
IL-13	0.720	0.597–0.833	0.00200	0.00240	0.450	1.000
IL-12 (p40)	0.700	0.574–0.820	0.00300	0.00343	0.775	0.577
TNF-α	0.593	0.459–0.722	0.09863	0.10760	0.275	1.000
IL-9	0.329	0.205–0.462	0.99300	0.99767	0.050	1.000
TNF-β	0.296	0.177–0.424	0.99767	0.99767	0.100	0.923

Values in red indicate statistical significance (p or q < 0.05).

### 4.4 ROC analysis in patients with acute pancreatitis

**Figure 5:**
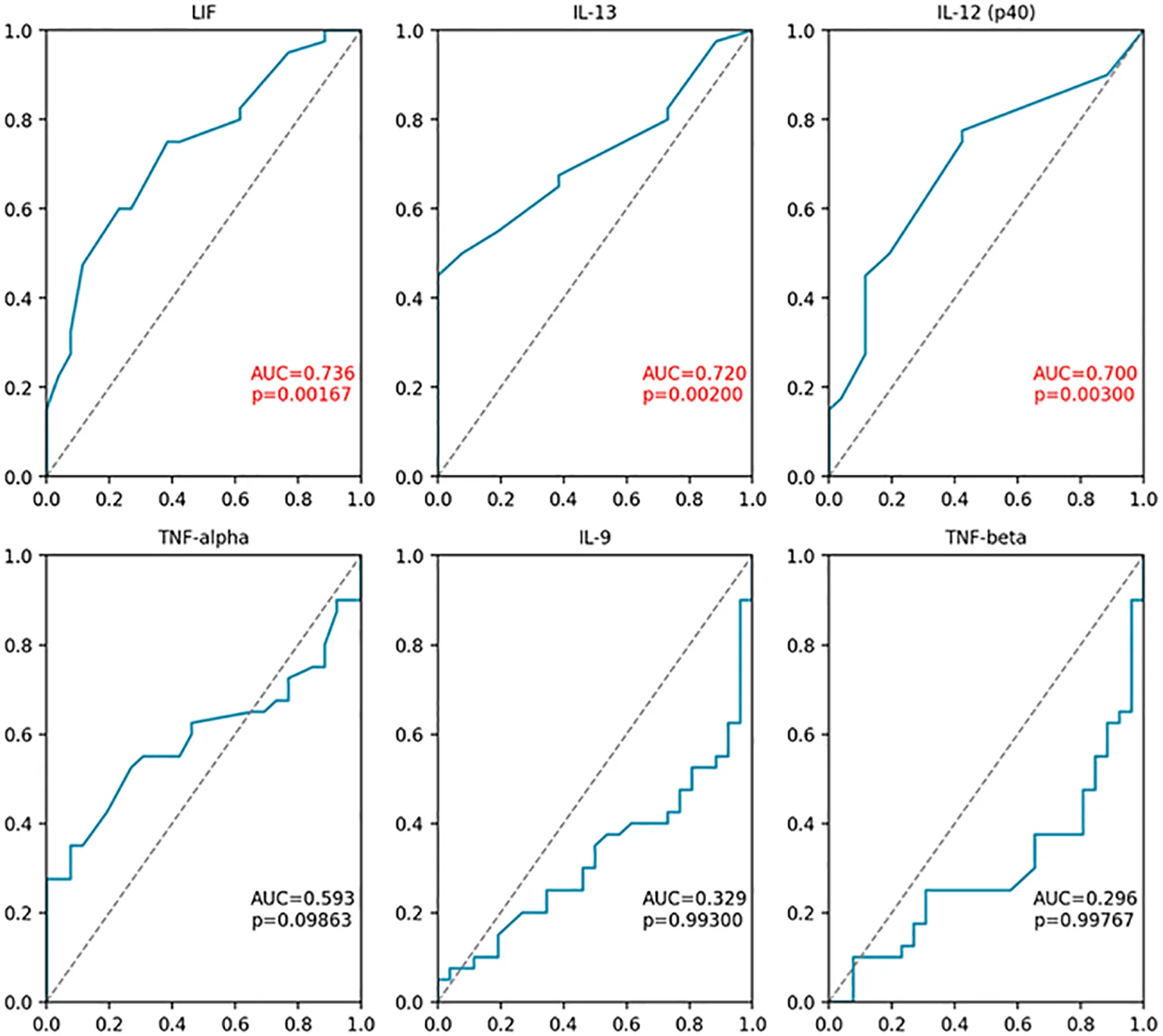
Receiver operating characteristic (ROC) analysis of cytokines of patients with acute pancreatitis and the control group.

**Figure 6:**
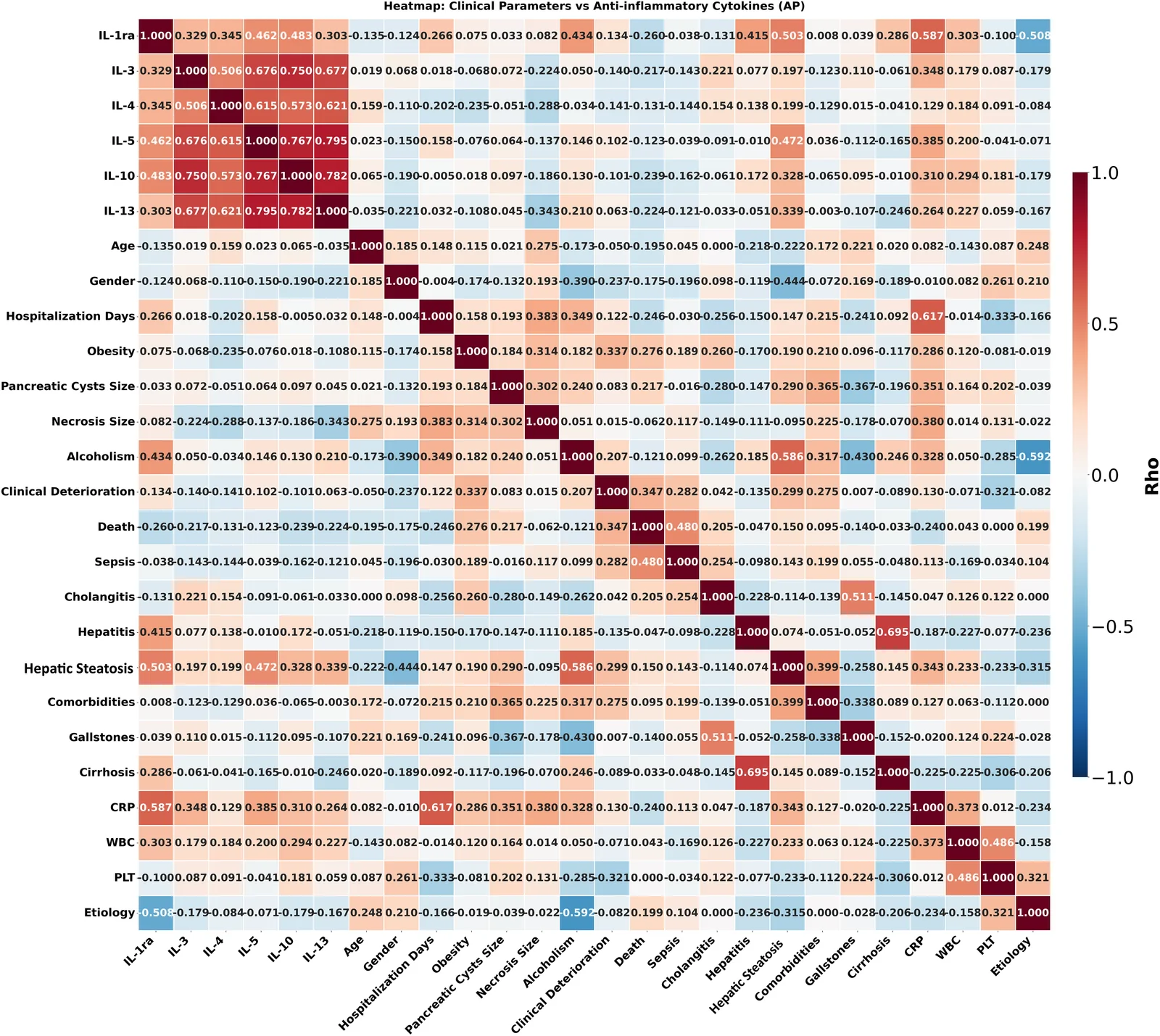
Receiver operating characteristic (ROC) analysis of cytokines of patients with acute pancreatitis and the control group.

Analysis of ROC curves revealed the highest diagnostic value for IL2Rα (AUC = 0.918) and IFNγ (AUC = 0.907). Both markers demonstrated statistical significance (p < 0.0001), which remained significant after FDR correction (q < 0.0001). Notably, IFNγ achieved perfect sensitivity (1.0) with a specificity of 0.694. Most other cytokines showed moderate diagnostic value (AUC 0.7 - 0.9). This group included IL16 (AUC = 0.899), IL1ra (AUC = 0.897), IL6 (AUC = 0.856), IL10 (AUC = 0.838), IL2 (AUC = 0.836), IL1β (AUC = 0.808), and IL1α (AUC = 0.806). We confirmed statistical significance (p < 0.05) for all listed parameters following correction for multiple comparisons. Within this cohort, we further distinguished markers with AUCs between 0.70 and 0.80, including MIF, IFNα2, IL3, IL4, IL17, IL12 (p70), LIF, IL18, and IL13. Interestingly, the panel also featured markers with limited or negligible diagnostic value. We observed limited value (AUC 0.5 - 0.7) for IL15 (AUC = 0.662), IL12 (p40) (AUC = 0.700), and TNFα (AUC = 0.593). For TNFα, the p-value was 0.09863 and yielded q = 0.10760 after FDR correction. In contrast, IL9 (AUC = 0.329) and TNFβ (AUC = 0.296) fell into the range defined as zero. P-values for these parameters exceeded 0.99, ruling out any diagnostic utility. Detailed results of the analyses of the diagnostic utility of inflammatory cytokines are presented in the **Table 4** and in **Figures 4B**–**6**.

**Figure 7:**
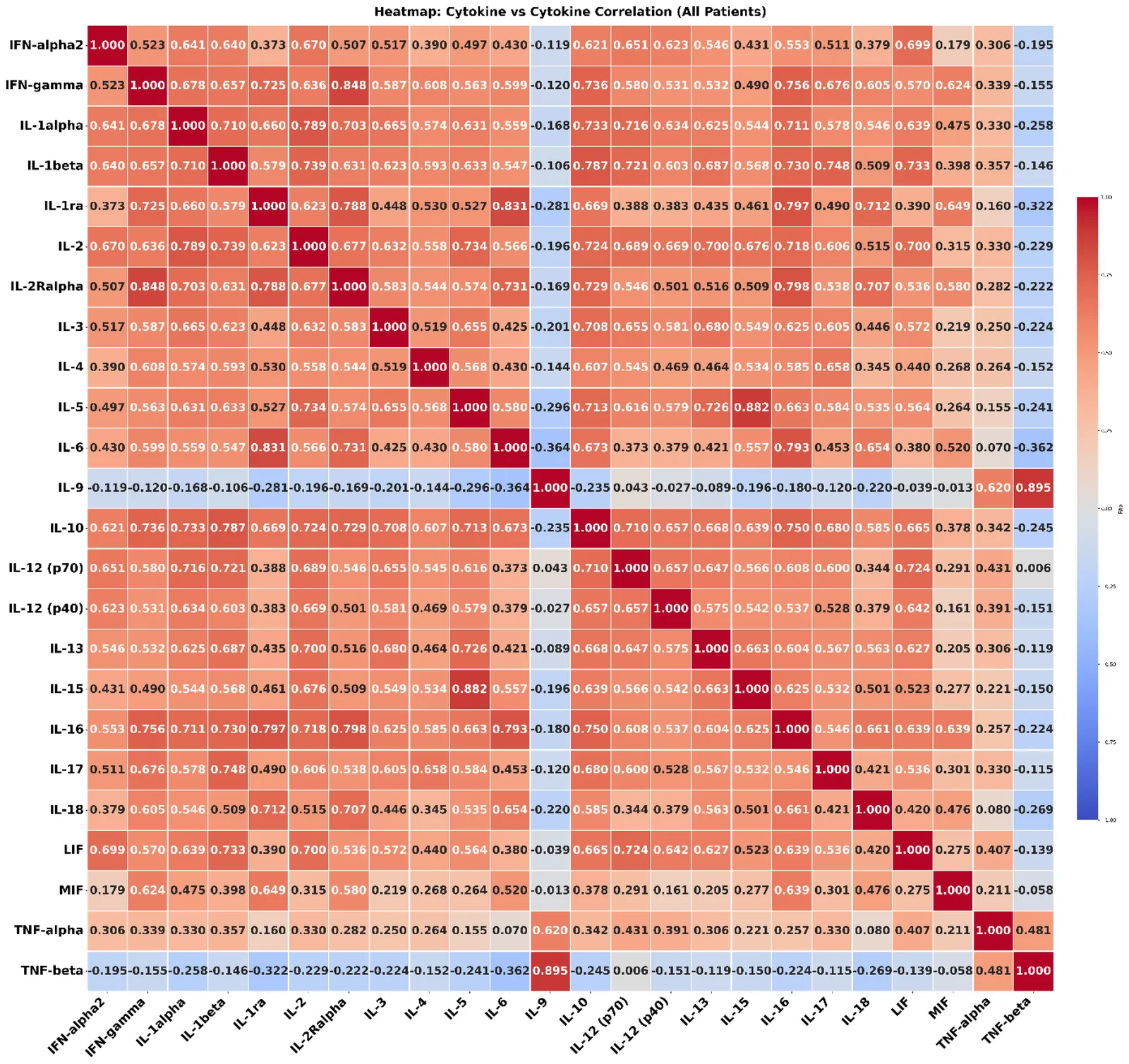
Heat map of correlations between all studied cytokines in patients with acute pancreatitis.

### 4.5 Correlations

**Figure 8:**
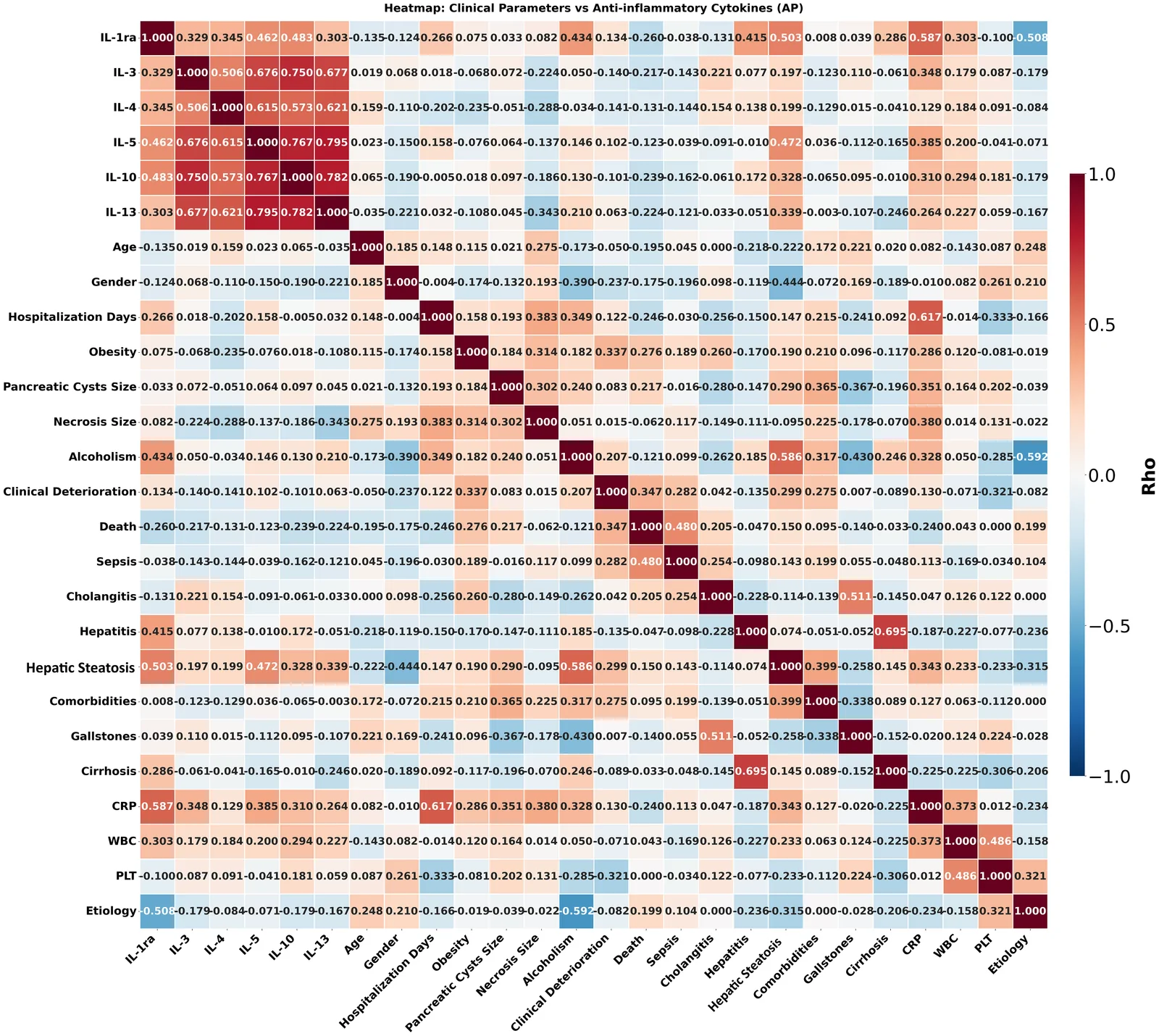
Heat map of correlations between anti-inflammatory cytokines and clinical parameters in patients with acute pancreatitis.

**Figure 9:**
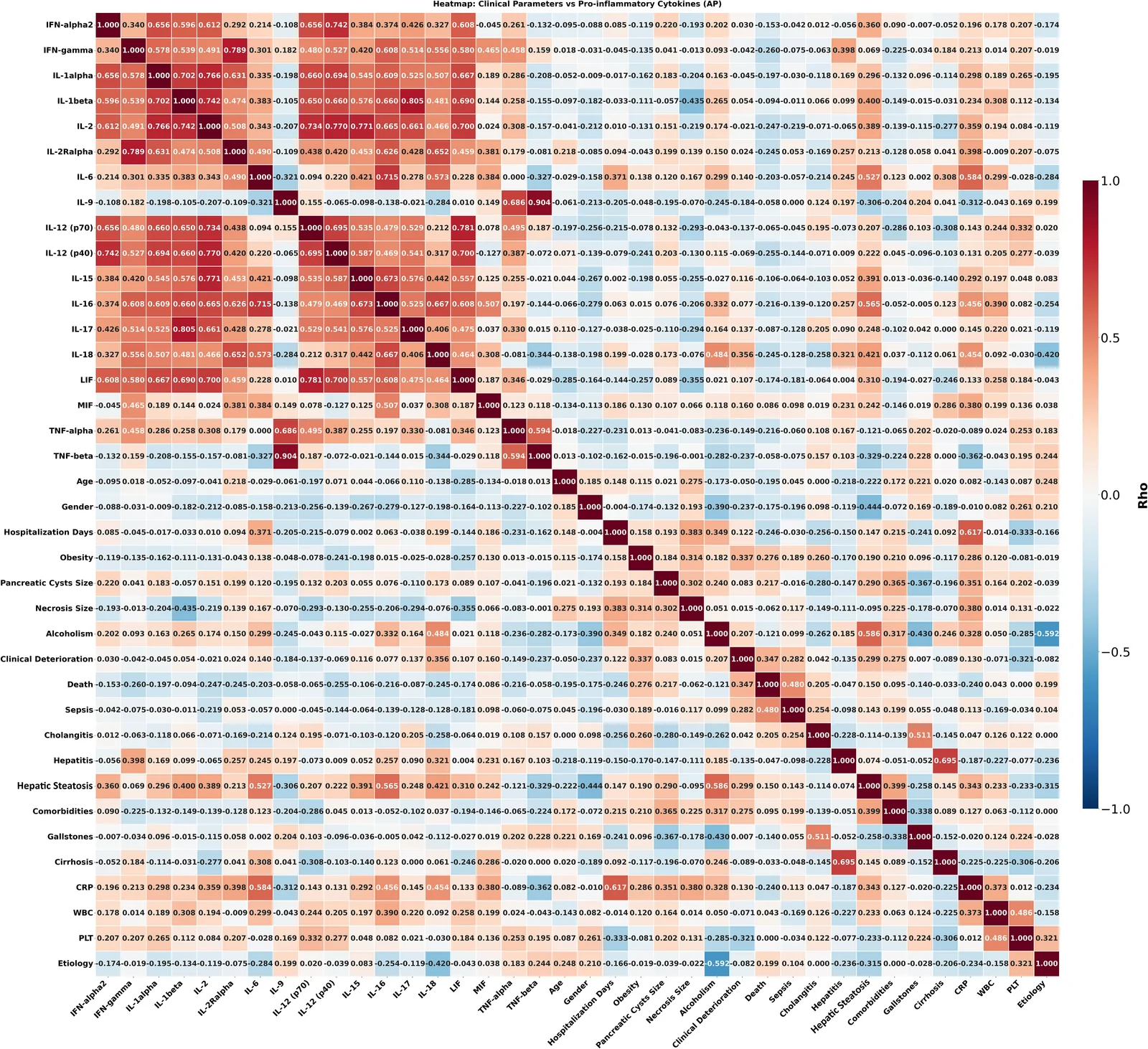
Heat map of correlations between pro-inflammatory cytokines and clinical parameters in patients with acute pancreatitis.

The correlation matrix revealed a clear, consistent pattern of positive relationships among most proinflammatory cytokines. Strongest associations involved markers along the IL-1 family, IL-6, IFN-γ. Strong correlation was observed between IL-1ra and IL-6 (ρ = 0.831). Within the IL-1 family high correlation was found between IL-1ra and IL-1β (ρ = 0.788). High rho values were reported between: IL-1α with IL-2 (ρ = 0.789) and IL-1β with IL-10 (ρ = 0.787). These links proved stable and independent of other markers, pointing to a shared regulatory component. Within the interferon group, we recorded a strong positive correlation between IFN-γ and IL-2Rα (ρ = 0.848) as well as IL-16 (ρ = 0.756), indicating parallel activation of cellular response pathways. IFN-α2 showed a similar but slightly weaker profile, with peaks at IL-2 (ρ = 0.670) and IL-12(p70) (ρ = 0.651). In the IL-1 axis, correlations between IL-1α, IL-1β, and IL-1ra were very strong and mutually consistent (all ρ > 0.65). IL-1ra also displayed one of the highest correlations in the entire matrix with IL-6 (ρ = 0.831), aligning with its role as a compensatory anti-inflammatory marker. Notably, IL-9 and TNF-β stood out for high variability, showing the only negative correlations. IL-9 correlated negatively with most proinflammatory markers (e.g., IL-6: ρ = -0.296), while TNF-β had the strongest negative ties with IL-1α (ρ = -0.258) and IL-6 (ρ = -0.362). Secondary markers like LIF and MIF showed moderate dependencies. LIF correlated positively with IL-1β (ρ = 0.733) and IL-12(p70) (ρ = 0.724), whereas MIF exhibited weak correlations with most other cytokines (mostly ρ < 0.40). All correlations are presented in heat maps (**Figures 7**–**9**); **Tables 5**, **6**.

**Table 5:** Spearman’s rank correlation coefficients between cytokine levels and clinical variables.

Cytokine	Variable	Rho	p
IFN-alpha2	Hepatic Steatosis	**0.360**	**0.024473**
IFN-γ	Hepatitis	**0.398**	**0.012081**
IL-10	Hepatic Steatosis	**0.328**	**0.041529**
IL-12 (p70)	Platelet count	**0.332**	**0.039065**
IL-13	Necrosis Size	**-0.343**	**0.030120**
IL-13	Hepatic Steatosis	**0.339**	**0.034569**
IL-15	Hepatic Steatosis	**0.391**	**0.013740**
IL-16	Alcoholism	**0.332**	**0.036525**
IL-16	Hepatic Steatosis	**0.565**	**0.000180**
IL-16	C-reactive protein	**0.456**	**0.003972**
IL-16	White blood cell count	**0.390**	**0.014005**
IL-18	Alcoholism	**0.484**	**0.001568**
IL-18	Clinical Deterioration	**0.356**	**0.026004**
IL-18	Hepatitis	**0.321**	**0.046544**
IL-18	Hepatic Steatosis	**0.421**	**0.007684**
IL-18	C-reactive protein	**0.454**	**0.004206**
IL-18	Etiology of Acute Pancreatitis	**-0.420**	**0.006917**
IL-1β	Necrosis Size	**-0.435**	**0.004979**
IL-1β	Hepatic Steatosis	**0.400**	**0.011734**

Only correlations reaching statistical significance (p < 0.05) are reported. Non-significant findings were excluded for clarity.

**Table 6:** Spearman’s rank correlation coefficients between cytokine levels and clinical variables.

Cytokine	Variable	Rho	p
IL-1rα	Alcoholism	**0.434**	**0.005134**
IL-1rα	Hepatitis	**0.415**	**0.008591**
IL-1rα	Hepatic Steatosis	**0.503**	**0.001092**
IL-1rα	C-reactive protein	**0.587**	**0.000106**
IL-1rα	Etiology of Acute Pancreatitis	**-0.508**	**0.000820**
IL-2	Hepatic Steatosis	**0.389**	**0.014456**
IL-2	C-reactive protein	**0.359**	**0.026956**
IL-2rα	C-reactive protein	**0.398**	**0.013306**
IL-3	C-reactive protein	**0.348**	**0.032512**
IL-5	Hepatic Steatosis	**0.472**	**0.002405**
IL-5	C-reactive protein	**0.385**	**0.017049**
IL-6	Hospitalization Days	**0.371**	**0.018396**
IL-6	Hepatic Steatosis	**0.527**	**0.000574**
IL-6	C-reactive protein	**0.584**	**0.000118**
LIF	Necrosis Size	**-0.355**	**0.024515**
MIF	C-reactive protein	**0.380**	**0.018589**
TNF-β	Hepatic Steatosis	**-0.329**	**0.040807**
TNF-β	C-reactive protein	**-0.362**	**0.025335**

Only correlations reaching statistical significance (p < 0.05) are reported. Non-significant findings were excluded for clarity.

Correlation analysis revealed multiple statistically significant relationships. The most numerous associations were observed for hepatic steatosis, which correlated positively with 10 cytokines, including IL-16 (r = 0.565, p = 0.00018), IL-6 (r = 0.527, p = 0.00057), and IL-1ra (r = 0.503, p = 0.00109). Other significant cytokines included IL-5 (r = 0.472, p = 0.002405), IL-18 (r = 0.421, p = 0.007684, IL-1β (r = 0.400, p = 0.011734), IL-2 (r = 0.389, p = 0.014456), IL-13 (r = 0.339, p = 0.034569, TNF-beta (r = -0.329, p = 0.040807), and IL-10 (r = 0.328, p = 0.041529). Regarding pancreatic necrotic lesions, significant negative correlations were found for IL-1 β (r = -0.435, p = 0.00498), LIF (r = -0.355, p = 0.02451), and IL-13 (r = -0.343, p = 0.03012). Alcoholism correlated positively with IL-18 (r = 0.484, p = 0.00157), IL-1ra (r = 0.434, p = 0.00513), and IL-16 (r = 0.332, p = 0.03652). Additionally, hepatitis was associated with IL-1rα (r = 0.415, p = 0.00859), and hospitalization duration with IL-6 (r = 0.371, p = 0.01839).

## 5 Discussion

Acute pancreatitis (AP) is a disease with a sudden onset of symptoms, primarily associated with severe pain in the epigastrium. It has been observed that in patients with AP, there is an intense release of inflammatory mediators. Accompanying the reaction is initial rapid recruitment and activation of cells of the innate immune response, including neutrophils and monocytes. In the case of a significant disturbance of the balance between pro- and anti-inflammatory mechanisms, damage to pancreatic tissue and distant organs may occur. If the inflammatory response is not limited appropriately and quickly, dangerous complications may develop. Among these are, for example, the formation of fluid cysts, necrotic changes of the pancreas and surrounding tissues, bacterial superinfections, or the development of multiple organ failure (5, 28–30). It has been confirmed that the severity of the inflammatory process in AP correlates with the risk of mortality and the patient’s long-term prognosis.

In the course of acute pancreatitis, inflammasome activation occurs (especially NLRP3), which is a key mechanism driving the inflammatory cascade, leading to organ necrosis and systemic complications. This sensor protein is associated with caspase-1 and caspase-8, the latter performing a proteolytic function toward pro-forms of, among others, IL-1β and IL-18 upon dimerization. During necrotic cell death, IL-1α is cleaved into its active form by caspases-5 and -11 (5, 28). Activation of additional caspases leads to the secretion of alarmin family proteins into the extracellular matrix, including TNF-α, IL-1α, IL-1β, IL-6, IL-8, and IL-10 (5). Alarmins exert multifactorial effects on the pancreatic extracellular matrix and modulate downstream stages of the inflammatory response (5, 29).

In our study, we demonstrated a statistically significant increase in cytokines: IL-1α, IL-1β, IL-6, IL-10 and other cytokines: IL-2Rα, IFN-γ, IL-1Rα, IL-16, IL-2, MIF, IFN-α, IL-5, IL-4, IL-3, IL-12 (p40), IL-12 (p70), IL-17, LIF, IL-18, IL-15, IL-13 as well as a decrease: TNF-β, IL-9. This indicates the secretion of both pro- and anti-inflammatory cytokines. The increase in concentrations of pro-inflammatory cytokines, such as IL-1α, IL-1β, IL-6, IFN-α, IFN-γ, IL-12 or IL-18, reflects the early activation of the innate immune response (30, 31). These reactions are initiated in response to cell damage and the release of DAMP, which is consistent with the observations of Malheiro et al. These researchers demonstrated elevated levels of IL-6, IL-10 and TNF-α already at the time of admission to the ward of patients with AP (30, 31). Similarly, Mititelu et al. described that IL-1β and IL-6 are key mediators initiating the NF-κB cascade and neutrophil recruitment in the first hours of the disease (30). At the same time, the decrease in TNF-β and IL-9, typically lymphocytic cytokines, indicates early lymphosuppression resulting from lymphocyte apoptosis, their migration to tissues, and transient inhibition of TCR signaling (32). This was described by Stojanovic et al. in the context of immunological dysregulation in severe inflammatory conditions (32).

Concurrently observed increase in anti-inflammatory cytokines, such as IL-10, IL-4, IL-13, IL-1Rα and LIF, confirms the activation of compensatory mechanisms aimed at limiting excessive inflammatory activation (33). IL-10, according to the results of York et al., Steen et al., inhibits the synthesis of pro-inflammatory cytokines by M1 macrophages and plays a key role in the modulation of inflammatory responses (34, 35). IL-4 and IL-13, as demonstrated by Scott et al., and Lundahl et al., block the Th1 response and direct macrophage differentiation towards the M2 phenotype with a repair-regulatory function (36, 37). As mentioned in the works of Kaplan et al. and Yuan et al., IL-1Ra, acts by competitive binding to IL-1R1, effectively inhibiting NF-κB activation (38, 39). LIF, on the other hand, as described by Jorgensen et al., serves as an early pleiotropic mediator with a predominance of anti-inflammatory action (40). The co-occurrence of these processes i.e. strong activation of the pro-inflammatory axis, parallel initiation of anti-inflammatory mechanisms, and early silencing of the lymphocytic response, highlights the complexity of the immunological reaction in the initial phase of AP. Clinically, this indicates the capture of the most dynamic stage of the disease. During this period, the balance between activation and regulation of the inflammatory response or its lack determines the further clinical course.

Among the evaluated analytes, IL-2Rα (AUC = 0.918) and IFN-γ (AUC = 0.907) demonstrated high discriminatory power for AP, retaining significance after FDR correction. IFN-γ achieved strong sensitivity at the selected cutoff, while IL-2Rα balanced high sensitivity with robust specificity. In contrast, IL-1Ra, IL-16, IL-6, IL-10, IL-2, IL-1β, and IL-1α exhibited moderate diagnostic value, whereas IL-9 and TNF-β lacked discriminative utility. The short half-life of TNF-α and its rapid receptor shedding may explain its limited utility despite its established pro-inflammatory role (41, 42). These results underscore that systemic cytokine dysregulation does not automatically translate into clinically actionable biomarkers, supporting the development of multianalyte panels that capture both innate and adaptive immune response.

Interestingly, no statistically significant differences in cytokine levels were observed with respect to the severity of acute pancreatitis. This finding may suggest that the analyzed cytokines are more strongly associated with the underlying etiology of the disease rather than its clinical severity. However, several factors should be considered when interpreting these results. First, the relatively small sample size may have limited the statistical power to detect subtle differences between severity groups. Second, the heterogeneous nature of the study population could have influenced cytokine variability. Finally, the timing of blood sample collection may have played an important role, as cytokine levels are known to change dynamically during the course of acute inflammation. Therefore, further studies with larger cohorts and standardized sampling protocols are needed to better elucidate the relationship between cytokine profiles and disease severity in acute pancreatitis.

The correlation analysis points to a clear relationship between the measured cytokine patterns and the patients’ clinical parameters. The strong link between IL-6, IL-16, and IL-1Ra and hepatic steatosis fits with a hepatobiliary-inflammatory pathway in AP (43, 44). This likely reflects either cytokines spilling into the circulation from the pancreas affected by inflammatory changes or shared metabolic dysfunction driving inflammation in both organs (33). Likewise, the association of IL-18 and IL-1Ra with alcohol use disorder suggests that these patients may already have a primed immune response, likely driven by alcohol-related tissue damage or disregulation in ROS balance (45, 46). We also noted an inverse relationship between IL-1β levels and severity of pancreatic necrosis. It suggests that a strong early IL-1β signal may have accelerated the recruitment of neutrophils and macrophages (47). In the later phase of acute pancreatitis, pancreatic and peripancreatic necrosis can become an important factor of ongoing sterile inflammation. The inverse association between IL-1β levels and necrosis likely reflects these temporal dynamics, with the early IL-1β peak occurring before significant necrotic injury has developed (48). This hypothesis should be confirmed in prospective studies with longitudinal monitoring, using repeated imaging assessments and tissue analysis.

Unlike conventional severity scores such as BISAP, Ranson, or APACHE II, which often rely on delayed or retrospective markers like BUN, hematocrit, and age, cytokine profiles capture the dynamic immune response during the initial hours of the disease. IL-2Rα and IFN-γ, in particular, could potentially serve as real-time indicators of early T-cell activation and macrophage polarization, making them strong candidates for early selection within the first 24 hours. Clinically, specific cytokine patterns may help in differentiating the course of the disease. AP profile with elevated IL-2Rα and IFN-γ alongside suppressed IL-1β could identify a high-risk subgroup prone to rapid pancreatic necrosis and multiorgan failure, warranting intensive monitoring and early intervention. Conversely, a balanced pro- and anti-inflammatory profile, characterized by robust IL-10, IL-4, and IL-13 levels, may indicate a milder clinical course, allowing for earlier de-escalation of care. Beyond risk stratification, these cytokine patterns directly inform therapeutic decisions. They could guide precision immunomodulation, such as early IL-1 blockades in IL-1β-dominant phenotypes or targeted neutralization of IFN-γ and TNF-α in hyperinflammatory states. Furthermore, serial cytokine monitoring enables dynamic treatment adjustment, helping clinicians intercept late complications like infected necrosis or progression to chronic pancreatitis. When integrated with standard clinical scoring systems, cytokine profiling can shift AP management from a reactive, uniform approach to a proactive, phenotype-driven strategy that reduces overtreatment in mild cases and prevents undertreatment in severe, rapidly progressing diseases.

This study has several limitations that reflect its exploratory character. First, it was conducted in a single center, which limits generalizability and requires validation in independent cohorts. Second, the study population mainly included patients requiring inpatient care, so the findings may not fully represent milder cases. Third, the sample size (n = 50 AP patients; n = 40 controls) is adequate for an initial biomarker screen, but larger datasets are needed to define diagnostic thresholds and capture disease heterogeneity. Fourth, cytokines were measured at a single time-point, which prevents assessment of their temporal dynamics. Fifth, clinical parameters such as severity, necrosis, liver steatosis, and alcohol use were evaluated as part of routine care; standardized phenotyping could refine these associations. Finally, age and sex were reported descriptively for both groups and were not included as covariates in the statistical analyses, as comprehensive demographic profiling was not the primary focus of this investigation. These factors underline the exploratory nature of the study and highlight the need for prospective, multicenter validation.

## 6 Conclusions

Acute pancreatitis is driven by a complex, layered immune response involving innate effector activation, inflammasome-mediated cytokine processing, and early adaptive T-cell engagement. In our cohort, the cytokine profile reflects simultaneous pro-inflammatory activation, compensatory anti-inflammatory responses, and early modulation of lymphocyte-derived mediators. IL-2Rα and IFN-γ emerged as the most accurate early diagnostic markers, likely reflecting initial T-cell receptor engagement and macrophage activation. The upregulation of IL-10, IL-4, IL-13, and IL-1Ra underscores the dynamic interplay between inflammatory amplification and regulation, which may influence disease trajectory and organ protection. The observed inverse relationship between IL-1β and pancreatic necrosis likely reflects temporal dynamics, as IL-1β peaks early in inflammation, while necrosis progresses over days. Integrating multiparametric cytokine profiling into routine care could enhance early risk stratification, guide severity-based management, and inform targeted immunomodulation. However, findings should be interpreted considering the single-center design, modest sample size, and single-time-point sampling. Prospective, multicenter studies with serial cytokine measurements and etiology-stratified cohorts are needed to validate clinical utility and optimize risk prediction algorithms.

## Data Availability

The original contributions presented in the study are included in the article/supplementary material. Further inquiries can be directed to the corresponding author.

## Glossary

AP: Acute Pancreatitis
APACHE II: Acute Physiology and Chronic Health Evaluation II
AUC: Area Under the Curve
Bio-Plex: Bio-Plex Multiplex System
BISAP: Bedside Index for Severity in Acute Pancreatitis
CI: Confidence Interval
CRP: C-Reactive Protein
Caspase: Cysteine-aspartyl proteases
DAMP: Damage-Associated Molecular Pattern
ELISA: Enzyme-Linked Immunosorbent Assay
FDR: False Discovery Rate
HL: Hodges-Lehmann estimator
IQR: Interquartile Range
IL: Interleukin
LIF: Leukemia Inhibitory Factor
LLOQ: Lower Limit of Quantification
LLM: Large Language Model
Luminex: Luminex xMAP platform
MAPK: Mitogen-Activated Protein Kinase
MIF: Macrophage Migration Inhibitory Factor
Me: Median
M1/M2: Macrophage polarization states
MWU: Mann–Whitney U test
N: Number
NK: Natural Killer cells
NF-κB: Nuclear Factor Kappa-Light-Chain-Enhancer of Activated B Cells
NLRP3: NOD-Like Receptor Protein 3 Family Pyrin Domain Containing 3
p40/p70: Interleukin-12 subunits
p/q: p-value/q-value
PRR: Pattern Recognition Receptor
ROC: Receiver Operating Characteristic
ROS: Reactive Oxygen Species
Ranson: Ranson’s criteria
SA-PE: Streptavidin Phycoerythrin
SIRS: Systemic Inflammatory Response Syndrome
TCR: T-cell receptor
Th1: T helper 1 cells
TNF: Tumor Necrosis Factor
WBC: White Blood Cell
xMAP: xMAP Technology (Luminex)
Youden Index: Youden’s J statistic index
α/β/γ: Cytokine subunit designations
δ: Cliff’s Delta (effect size)
%: Percentage
ρ: Spearman’s rank correlation coefficient
r: Correlation coefficient
